# Association between depression, anxiety and weight change in young adults

**DOI:** 10.1186/s12888-019-2385-z

**Published:** 2019-12-16

**Authors:** Berhe W. Sahle, Monique Breslin, Kristy Sanderson, George Patton, Terence Dwyer, Alison Venn, Seana Gall

**Affiliations:** 1 Menzies Institute for Medical Research, University of Tasmania, Private Bag 23, Hobart, Tasmania 7001 Australia; 2 School of Health Sciences, Faculty of Medicine and Health Sciences, University of East Anglia, Norwich, UK; 3 Centre for Adolescent Health, Murdoch Children’s Research Institute, Melbourne, Australia; 4 George Institute, University of Oxford, Oxford, UK

**Keywords:** Anxiety, Depression, Mood disorders, Weight change, BMI, Longitudinal

## Abstract

**Background:**

To investigate whether there are bi-directional associations between anxiety and mood disorders and body mass index (BMI) in a cohort of young adults.

**Methods:**

We analysed data from the 2004–2006 (baseline) and 2009–2011 (follow-up) waves of the Childhood Determinants of Adult Health study. Lifetime DSM-IV anxiety and mood disorders were retrospectively diagnosed with the Composite International Diagnostic Interview. Potential mediators were individually added to the base models to assess their potential role as a mediator of the associations.

**Results:**

In males, presence of mood disorder history at baseline was positively associated with BMI gain (β = 0.77, 95% CI: 0.14–1.40), but baseline BMI was not associated with subsequent risk of mood disorder. Further adjustment for covariates, including dietary pattern, physical activity, and smoking reduced the coefficient (β) to 0.70 (95% CI: 0.01–1.39), suggesting that the increase in BMI was partly mediated by these factors. In females, presence of mood disorder history at baseline was not associated with subsequent weight gain, however, BMI at baseline was associated with higher risk of episode of mood disorder (RR per kg/m^2^: 1.04, 95% CI: 1.01–1.08), which was strengthened (RR per kg/m^2^ = 1.07, 95% CI: 1.00–1.15) after additional adjustment in the full model. There was no significant association between anxiety and change in BMI and vice-versa.

**Conclusion:**

The results do not suggest bidirectional associations between anxiety and mood disorders, and change in BMI. Interventions promoting healthy lifestyle could contribute to reducing increase in BMI associated with mood disorder in males, and excess risk of mood disorder associated with BMI in females.

## Background

Mental disorders, including mood disorders and anxiety disorders, and obesity or overweight are very prevalent and associated with many other diseases. Globally, the prevalence of overweight or obesity exceeds 37% among adults aged 18 years and over, and has tripled over the last four decades [1]. The global lifetime prevalence of mood disorder and anxiety disorders is estimated to be 13 and 10%, respectively [2]. The number of people affected by mood disorders and anxiety has also increased worldwide by 18% and by 15%, respectively between 2005 and 2015 [3]. Mental disorders and overweight or obesity are frequently co-occurring conditions [4, 5], and share common risk factors such as lower socioeconomic status, physical inactivity, smoking, and alcohol consumption [6, 7]. These conditions have shared consequences, including increased risk of morbidity and mortality from several diseases such as cardiovascular diseases, type 2 diabetes, and certain types of cancer [8–11].

Depressive-anxiety disorders may contribute to weight gain through their association with poor eating habits, physical inactivity, and poor adherence to recommended lifestyle modifications, which could influence future changes in adiposity [6, 12, 13]. On the other hand, social stigma associated with excess weight or obesity may have a negative impact on body image, self-esteem, and social interactions that are related to the development of depressive-anxiety disorders [12, 13]. Medications used to treat several mental disorders are known to cause weight gain [14]. The link between depressive-anxiety disorders and change in body weight may also be confounded or mediated by several variables including age, gender and socioeconomic status [13, 15, 16]. Previous studies have shown that inflammation is associated with both weight change and mental health disorders, although the associations vary based on adjusting for confounders and on how inflammation and mental health disorders were measured [17–19]. Evidence also noted sex difference in the association between BMI and depression, and suggested for sex-specific analyses of both BMI and anxiety or depression [20]. Studies with consideration of a wide range of potential confounding or mediating variables are of use as they can help our understanding of the mechanisms for the associations and inform prevention and treatment strategies.

Mood disorders and anxiety, and weight gain are closely related and recognized as common conditions among adolescents and young adults [1, 7]. Most of the studies examining the link between depressive and anxiety disorders and body weight are cross-sectional, making it difficult to discern the temporal relationship of these conditions. Longitudinal studies that examined the direction of the association between depressive-anxiety disorders and change in body weight have mixed findings; with meta-analyses and large cohort studies showing that some [21–23], but not all studies [15, 24, 25] report bidirectional associations between these conditions. Evidence of temporal sequences mood disorders and anxiety, and weight change among young adults can help to optimize the prevention, early detection, and treatment services for both disorders. This study investigated whether there are bi-directional associations between anxiety and mood disorders, and BMI change in a prospective cohort of young adults.

## Methods

### Study design and participants

**Fig. 1:**
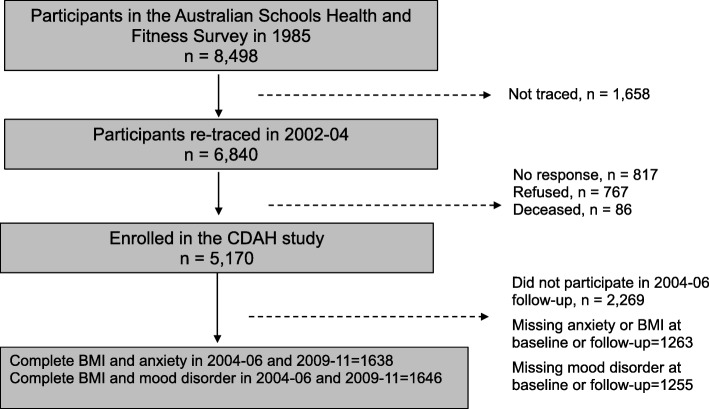
Participation flow chart

The present study analysed data from the Childhood Determinants of Adult Health (CDAH) study. Details of the study design and participants were previously reported [26, 27]. In brief, the CDAH study started with the follow-up of 8498 schoolchildren (7–15 years) who participated in the 1985 Australian Schools Health and Fitness Survey (ASHFS) [27]. The CDAH study was set up to investigate the contribution of childhood factors to the risk of developing cardiovascular diseases in later life [27]. At baseline (2004–2006), and at follow-up (2009–2011) participants completed assessments of mood disorder, anxiety disorders and their lifestyle behaviours. The present study included 11,638 participants who have complete anxiety and BMI data at baseline and at follow-up, and 1646 who have complete baseline and follow-up data on BMI and mood disorder (Fig. 1) [28]. Participants were 26–36 years at baseline and 31–41 years at follow-up.

### Covariates

At baseline and follow-up, participants reported recent sociodemographic information, including occupation, education, and marital status. Details of the baseline and follow-up measurements and procedures have been described previously [26, 28]. Family history of cardiometabolic disease (myocardial infarction, stroke, high blood pressure, coronary heart disease and diabetes), lifestyle behaviours and risk factors, including smoking, and alcohol consumption were recorded using self-administered questionnaire [26].

When completing the Food Frequency and Habits questionnaire, participants were asked to refer to their diet during the 12 months prior to the interview. The combined Dietary Guideline Index (DGI) was used to generate a dietary score. It assesses adherence to the Dietary Guidelines for Australian Adults and covers consumption of foods from the core food groups, along with other items such as use of salt and fluid intake. The DGI includes 15 items that are scored using 127 items from a food frequency questionnaire [12]. Higher scores indicated closer adherence to dietary guidelines. The International Physical Activity Questionnaire (IPAQ) was used to assess the duration of moderate- and vigorous-intensity leisure, work, active commuting, and yard or household physical activity in the past week. Total physical activity is calculated by summing all four domain sub-scores according to the IPAQ scoring protocol [29].

Residential postcode was used to derive area-level socio-economic status based on the Australian Bureau of Statistics Postal Area Index of Relative Socio-Economic Disadvantage. The Henderson Interview Schedule for Social Interaction (ISSI) asks respondents about the number of people with whom they share strong affectual attachments and about the perceived adequacy of these relationships were used to derive a Social Support Index (SSI) [30]. We used the NEO Five-Factor Inventory (NEO-FFI) to assess the personality (Neuroticism, Extraversion, Openness to Experience, Agreeableness, and Conscientiousness) of the participants [31, 32].

Serum C-reactive protein (CRP) at baseline was measured from fasting venous blood samples using an automated analyser and a highly sensitive turbidimetric immunoassay kit. Fasting plasma glucose was measured enzymatically with the Olympus AU5400 automated analyser. Blood pressure was measured at follow-up using a digital automatic monitor.

At baseline, BMI was calculated as weight divided by the square of height. For those with self-reported height and weight data, a correction factor was applied based on a validation study from the same cohort [33]. At follow-up, all participants reported their BMI, and therefore the correction factor applied to all BMI. We classified persons into three BMI categories: Normal weight (18.50–24.99), overweight (25 ≤ BMI ≤ 29.99) and obese (BMI ≥ 30).

### Assessment of anxiety and mood disorders

At follow-up, lifetime history of DSM-IV disorder were obtained from the Composite International Diagnostic Interview (CIDI-Auto 2.1 version) via telephone interview with trained interviewers [26].

“Presence of mood disorder history at baseline” was defined as an episode of depression/dysthymia that occurred prior to baseline; and “presence of anxiety history at baseline” as an episode of anxiety that occurred prior to baseline. We categorized those with a diagnosis of mood disorders, or anxiety disorder between baseline and CDAH2 as having ‘episode’ (first or recurrent events) of these disorders, or having ‘first episode’ in those without history mood or anxiety disorders who suffered from these disorders for the first time since baseline.

### Statistical analyses

#### History mood and anxiety disorder predicting BMI change

We used linear regression models to estimate the association of history of mood disorders before baseline and BMI change measured as body mass index (BMI) at follow-up adjusted for potential confounders including BMI at baseline. This method, sometimes known as repeated measures analysis of covariance, is a preferred method for analyses of longitudinal changes with only two time-points [34, 35]. We used the same approach to examine if history of anxiety disorder predicted subsequent BMI change.

#### BMI at baseline predicting mood and anxiety disorders

We used log-binomial regressions to estimate the relative risks (RR) ± 95% confidence interval (CI) of baseline BMI (per 1 kg/m^2^) on mood and anxiety disorders diagnosed since baseline, while adjusting for potential confounders. Sensitivity analyses were conducted by limiting these associations to those who had an episode of mood and anxiety disorder diagnosed in the 12 months prior to CDAH2. Approaches to the data analyses and variables included in the base model are given in the Additional file 1. Both analyses were stratified by sex because of presumed differences in the relationship between BMI and mental health disorders.

In the base model, the associations were adjusted for potential confounders, including age, history of cardiometabolic conditions, social support and duration of follow-up. Other covariates including C-reactive protein, antidepressant use, dietary pattern and lifestyle factors were serially added to the base models to test their potential role as a mediator of the associations. In the linear regression models, the mediation effect was calculated as the difference between the total effect (base model without mediator) and the direct effect (base model with mediator) [36]. In the log-binomial regressions models, the percentage of excess risk explained by the mediator (PERM) was estimated as a ratio of the difference between the unadjusted (total effect) and the adjusted (direct effect) relative risks, and the unadjusted excess risk (total effect) [37], as:

$$ \mathrm{PERM}=\frac{\mathrm{Confounder}\ \mathrm{adjusted}\ \mathrm{RR}-\mathrm{Confounder}\ \mathrm{and}\ \mathrm{mediator}\ \mathrm{adjusted}\ \mathrm{RR}\ }{\mathrm{Confounder}\ \mathrm{adjusted}\ \mathrm{RR}-1}\ast 100. $$

#### Follow-up and handling of missing data

For the current study, 3965 participants completed questionnaires at baseline about their health behaviours and outcomes, including those who undertook physical measures of the cardiovascular health in clinics (*n* = 2385). At follow-up, 2815 of the eligible sample at baseline completed questionnaires. Out of these, 1646 participants (*n* = 1012 females) who had complete data on BMI and presence of mood disorder history at baseline and at follow-up were included in the analyses of mood disorder and BMI change and vice-versa. The analyses on anxiety and BMI change included 1638 participants (1008 females) who had complete data for those variables at baseline and at follow-up and vice-versa.

For the sample with complete outcome and predictor data as described above, we imputed any missing values of covariates using multiple imputation by chained equations (with 50 estimates). Variables recorded at the original childhood study as well as baseline and follow-up that predicted probability of response were included in the missingness model, including sociodemographics, fitness and health behaviours. We used inverse probability weights for addressing loss from the original ASHFS random sample to the analysis sample. Any observations required for the weights model that were missing were imputed using the imputed datasets, so that a complete set of weights for the analysis sample was available. For each of the fifty imputed datasets a set of weights was derived and applied to the analysis model. The resulting fifty different model estimates were combined using Rubin’s rules to get an average point estimate and standard error that reflects variation in the weights and imputed covariates, as well as the individual estimates.

## Results

Table 1 presents a summary of participant characteristics. In general, about half of the participants were from low or medium to low socioeconomic status, and nearly half had tertiary education at follow-up. History of heart diseases or diabetes was reported by 16% of males and 10% of females. History of mood and anxiety disorder were nearly twice as common in females as males both at baseline and at follow-up.

**Table 1:** Characteristics of the study participants included in the analyses

Variables	Mood disorder before baseline predicting BMI (*n* = 1646) ^#^		Anxiety before baseline predicting BMI (*n* = 1638) ^#^	
	Men (*n* = 634)	Women (*n* = 1012)	Men (*n* = 630)	Women (*n* = 1008)
Age, years	31.9 ± 2.6	31.6 ± 2.7	31.9 ± 2.6	31.6 ± 2.7
Education				
Tertiary	282 (44.7)	520 (51.8)	280 (44.7)	517 (51.8)
Vocational	235 (37.2)	269 (26.8)	234 (37.3)	268 (26.8)
School only	114 (18.1)	214 (21.3)	113 (18.0)	214 (21.4)
Socio-economic quartile				
High	742 (23.2)	748 (24.1)	134 (26.2)	211 (27.1)
Med-high	914 (28.6)	886 (28.6)	141 ()27.6	209 (26.9)
Med-low	1230 (38.5)	1197 (38.6)	196 (38.4)	314 (40.4)
Low	310 (9.7)	272 (8.8)	40 (7.8)	44 (5.7)
Smoking				
Never	385 (61.0)	582 (57.6)	384 (61.2)	580 (57.6)
Former	141 (22.4)	295 (29.2)	140 (22.3)	294 (29.2)
Current	105 (16.6)	134 (13.2)	103 (16.4)	133 (13.2)
Change in marital status since baseline				
Never married	160 (14.1)	179 (11.5)	75 (13.0)	116 (12.5)
Become married/defacto	179 (15.7)	170 (10.9)	99 (17.2)	75 (8.1)
Stayed married	741 (65.1)	1097 (70.4)	381 (66.0)	674 (72.7)
Became divorced/widowed	32 (2.8)	56 (3.6)	10 (1.7)	29 (3.1)
Stayed separated/widowed	8 (0.7)	24 (1.5)	4 (0.7)	13 (1.4)
Parental status since baseline				
No children	436 (34.60)	484 (28.2)	206 (40.0)	265 (27.2)
First child born	315 (25.0)	326 (19.0)	164 (27.1)	185 (19.0)
Additional children born	235 (18.7)	359 (20.9)	110 (18.2)	214 (22.0)
Same number of children	274 (21.8)	548 (31.9)	126 (20.8)	311 (31.9)
Body mass index (kg/m^2^)	26.6+ 4.4	25.5 ± 5.7	26.6 ± 4.5	25.5 ± 5.7
History of CVD or diabetes	83 (16.0)	83(9.9)	83 (16.1)	83 (10.0)
Social support index	*N* = 543, 61.2 ± 8.0	*N* = 872, 62.5 ± 7.1	61.3 ± 8.0	62.6 ± 7.1
Total healthy lifestyle score	2.2 ± 1.8	2.2 ± 1.7		
SF-12 physical component score	53.7 ± 6.4	53.2 ± 7.6	53.7 ± 7.5	53.3 ± 7.5
Total diet score	99.7 ± 19.1	107.9 ± 17.4	99.8 ± 19.2	107.8 ± 17.4
Anxiety before baseline	77 (12.0)	210 (19.1)	76 (12.1)	195 (19.4)
Mood disorder before baseline	85 (13.4)	229 (22.6)	85 (13.5)	226 (22.4)
Lifetime depression (at CDAH2)				
Never	521 (82.2)	710 (70.2)	532 (84.4)	751 (74.5)
History	30 (4.7)	102 (10.1)	16 (2.5)	27 (2.7)
Recurrent	55 (8.7)	127 (12.6)	60 (9.5)	168 (16.7)
Incident	28 (4.4)	73 (7.2)	22 (3.5)	62 (6.5)
Mood disorder 12 months prior to CDAH2 (incident and recurrent)	40 (6.3)	92 (9.1)	40 (6.3)	91 (9.0)
Lifetime anxiety (at CDAH2)				
Never	532 (84.4)	751(74.5)	517(82.1)	709 (70.3)
History	16 (2.5)	27 (2.7)	30 (4.8)	100 (9.9)
Recurrent	60 (9.5)	168 (16.7)	55 (8.7)	126 (12.5)
Incident	22 (3.5)	62 (6.2)	28 (4.4)	73 (7.2)
Anxiety 12 months prior to CDAH2 (incident and recurrent)	47 (7.5)	141 (14.0)	47 (8.1)	141 (15.8)

*N (%)* Mean ± standard deviation, *CDAH* Childhood Determinants of Adult Health, *CVD* Cardiovascular diseases (Myocardial infarction, stroke, coronary heart disease, high blood pressure), *SF-12* Short Form 12, a health-related quality of life instrument

### History of mood disorder and anxiety predicting BMI change

Of the total 634 males and 1012 females who have complete data for BMI and presence of mood disorder history at baseline and at follow-up, 85 (13.4%) males and 229 (22.6%) females had a history of mood disorder at baseline. In males, after adjustment for covariates in the base model, including age, education, family history of cardiometabolic diseases and social support score, history of mood disorder at baseline was associated with an increase in BMI (β = 0.77, 95% CI: 0.14–1.40, *P* = 0.017). Serial adjustment for potential mediators, including smoking status, adherence to DGI, moderate-to-vigorous physical activity reduced the average BMI increase due to history of mood disorder to 0.70 (β = 0.70, 95% CI: 0.01–1.39). This suggests that 0.07 kg/m^2^ of the average increase in BMI is mediated by these factors. However, antidepressant use has no effect on the association between history of mood disorder and BMI change (β = 0.77, 95% CI: 0.05–1.49). In males, extra food consumption, moderate or vigorous physical activity, dietary adherence and smoking status appeared to be the strongest mediators (Table 2).

**Table 2:** Association between history of mood disorders before baseline and BMI at follow-up

Variables	β (95% CI) after inclusion of potential mediators or confounders^a^	Total mediated effect
Males		
Baseline measurements		
Base model	0.77 (0.14–1.40)	**–**
C-reactive protein	0.67 (−0.02–1.37)	0.10
Moderate or vigorous physical activity	0.85 (0.14–1.56)	−0.08
Steps per day	0.72 (0.03–1.42)	0.05
Dietary adherence	0.70 (0.01–1.39)	0.07
Dietary guideline index	0.71 (0.02–1.4)	0.06
Extra food consumption	0.88 (0.11–1.64)	−0.11
Takeaway food consumption	0.70 (0.01–1.39)	0.07
Antidepressant use	0.77 (0.05–1.49)	0
Smoking status	0.70 (0.02–1.38)	0.07
Additional follow-up variables		
Moderate or vigorous physical activity	0.70 (0.00–1.40)	0.07
Steps per day	0.68 (−0.01–1.40)	0.09
Dietary guideline index	0.71 (0.01–1.40)	0.06
Dietary adherence	0.71 (0.02–1.40)	0.06
Change between baseline and follow-up		
Change in steps per day	0.68 (−0.02–1.38)	0.09
Change in smoking status	0.72 (0.04–1.39)	0.05
Change in dietary Guideline Index	0.70 (0.01–1.39)	0.07
Full model	0.70 (0.01–1.39)	0.07
Females		
Baseline measurements		
Base model	0.53 (0.00,1.06)	**–**
C-reactive protein	0.41 (−0.14,0.96)	0.12
Moderate or vigorous physical activity	0.41 (−0.15,0.96)	0.12
Steps per day	0.41 (−0.14,0.97)	0.12
Dietary adherence	0.40 (−0.15,0.95)	0.13
Dietary Guideline Index	0.40 (−0.15,0.95)	0.13
Extra food consumption	0.59 (0.02,1.16)	−0.06
Takeaway food consumption	0.41 (−0.14,0.96)	0.12
Antidepressant use	0.46 (−0.16,1.07)	0.07
Smoking status	0.39 (−0.17,0.95)	0.14
Additional follow-up variables		
Moderate or vigorous physical activity	0.40 (−0.15,0.95)	0.13
Steps per day	0.34 (−0.21,0.90)	0.19
Dietary Guideline Index	0.40 (−0.15,0.95)	0.13
Dietary adherence	0.40 (−0.15,0.96)	0.13
Change between baseline and follow-up		
Change in steps per day	0.39 (−0.16,0.94)	0.14
Change in smoking status	0.44 (−0.11,0.99)	0.09
Change in dietary Guideline Index	0.39 (−0.16,0.95)	0.14
Full model	0.39 (−0.16,0.95)	0.14

Base model includes: Baseline BMI (kg/m2), age, education, duration of follow-up, history of cardiometabolic disease, Social Support index, physical activity, self-reported physical health status and extraversion

^a^Covariates, associated with mood disorder based on a univariate models (at *p* < 0.1) were added one at a time to the base model to test for mediating

Among females, a history of mood disorder was associated with a smaller increase in BMI (β = 0.53, 95% CI: 0.00–1.06, *p* = 0.048), which was attenuated (β = 0.39, 95% CI: − 0.16-0.95) after further adjustment for participants health behaviours. The full list of the effect of individual covariates on the associations between history of mood disorder and BMI change in males and females is presented in Table 2*.*

In the base model, history of anxiety was associated with a non-significant increase in BMI in males (β = 0.16, 95% CI: − 0.45-0.76) and females (β = 0.01, 95% CI: − 0.59-0.61). Further adjustment for potential mediators, including C-reactive protein, antidepressant use, physical activity, diet and smoking did not significantly change the associations between history of anxiety and BMI change in both males (β = 0.19, 95% CI: − 0.44-0.62) and females (β = 0.16, 95% CI: − 0.48-0.80). The results are presented as Additional file 2.

### Baseline BMI predicting mood and anxiety disorders

In females (RR per kg/m^2^: 1.04, 95% CI: 1.01–1.08), BMI at baseline tended to increase the risk of episode of mood disorder between baseline and follow-up, and mood disorder diagnosed in the 12 months prior to CDAH2 (RR per kg/m^2^: 1.03, 95% CI: 1.00–1.07). Serial adjustment strengthened both the risk of mood disorder (RR per kg/m^2^: 1.07, 95% CI: 1.00–1.15) and risk of mood disorder diagnosed in the 12 months prior to CDAH2 (RR per kg/m^2^: 1.06, 95% CI: 1.00–1.13) (Table 3).

**Table 3:** Effect of potential confounders or mediators on the associations between BMI at baseline and episode of mood disorder

Variables	Episode of mood disorder since baseline (vs. those with no history)	PERM	Episode of mood disorder 12 leading to CDAH2 (vs those with no history)	PERM
	Adjusted RR (95% CI) after inclusion of covariate		Adjusted RR (95% CI) after inclusion of covariate	
Males				
Base model	0.97 (0.90,1.04)		0.94 (0.86,1.03)	–
C-reactive protein	0.94 (0.82,1.08)	−100	0.92 (0.84,1.02)	−33.3
Dietary guideline index	0.95 (0.87,1.03)	−66.7	0.94 (0.85,1.03)	0
Antidepressant use	0.92 (0.83,1.03)	− 166.7	0.94 (0.85,1.04)	0
Smoking status	0.94 (0.86,1.03)	100	0.94 (0.85,1.04)	0
Fibrinogen	0.91 (0.88,1.06)	200	0.94 (0.85,1.04)	0
Moderate or vigorous physical activity	0.88 (0.68,1.12)	300	0.88 (0.72,1.07)	−100
Steps per day	0.89 (0.69,1.15)	− 266.7	0.88 (0.72,1.08)	−100
Extra food consumption	0.94 (0.83,1.07)	−100	0.94 (0.85,1.04)	0
Smoking status at follow-up	0.92 (0.81–1.06)	−166.7	0.87 (0.72,1.07)	− 116.7
Moderate or vigorous physical activity	0.88 (0.64,1.20)	300	0.86 (0.70,1.06)	− 133.3
Dietary guideline index	0.94 (0.80–1.09)	−100	0.93 (8.83–1.04)	−16.7
Weight satisfaction	0.90 (0.76,1.08)	− 233.0	0.92 (0.80,1.07)	−33.3
Dietary adherence	0.86 (0.61,1.21)	− 366.0	0.87 (0.70,1.09)	−116.7
Takeaway food consumption	0.93 (0.78,1.11)	−133.3	0.93 (0.83,1.04)	−16.7
Full model	0.93 (0.78,1.11)	−133.3	0.93 (0.83,1.04)	−16.7
Females				
Base model	1.04 (1.01,1.08)		1.03 (1.00,1.07)	–
C-reactive protein	1.09 (1.01,1.17)	− 125.0	1.06 (1.03,1.11)	−100
Dietary guideline index	1.05 (1.00,1.11)	−25.0	1.06 (1.02,1.10)	−100
Antidepressant use	1.06 (0.99,1.14)	−50.0	1.07 (1.02,1.12)	−133.3
Smoking status	1.07 (1.02,1.13)	−75.0	1.06 (1.02,1.10)	−100
Fibrinogen	1.13 (0.98–1.30)	− 225	1.06 (1.01–1.11)	− 100
Moderate or vigorous physical activity	1.13 (0.98,1.30)	−225	1.12 (1.02,1.23)	− 300
Steps per day	1.15 (0.99,1.33)	275	1.13 (1.00,1.28)	− 333.3
Extra food consumption	1.07 (1.00,1.15)	−75.0	1.06 (1.00,1.13)	−100
Smoking status at follow-up	1.06 (0.99–1.14)	−50.0	1.12 (1.02,1.23)	−300
Moderate or vigorous physical activity at follow-up	1.06 (0.99,1.14)	−50.0	1.06 (1.01,1.11)	−100
Dietary guideline index at follow-up	1.13 (0.98–1.30)	−225.0	1.13 (1.02–1.24)	−333.3
Weight satisfaction	1.08 (1.00,1.16)	−100	1.11 (0.94,1.30)	266.7
Dietary adherence	1.15 (0.99,1.33)	− 275.0	1.07 (1.00,1.13)	−133.3
Takeaway food consumption	1.07 (1.00,1.15)	−75.0	1.06 (1.00,1.13)	−100
Full model	1.07 (1.00,1.15)	−75.0	1.06 (1.00,1.13)	−100

*RR* relative risk, *CI* confidence interval, Base model adjusted for: Age, education, duration of follow-up, marital status, self-reported physical health status, history of cardiometabolic diseases, and use of oral contraceptive (in women), *PERM* percentage of excess risk explained by the mediator

In males, BMI at baseline did not predict the risk of mood disorder (RR per kg/m^2^: 0.97, 95% CI: 0.90–1.04), or mood disorder diagnosed in the 12 months prior to CDAH2 (RR per kg/m^2^: 0.94, 95% CI: 0.86, 1.03) in males. These findings persisted after adjustment for potential mediators including health behaviours, diet and physical activity. In females, adjusting for body weight satisfaction increased the risk of an episode of mood disorder (RR per kg/m^2^: 1.08 95% CI: 1.00–1.16) but not mood disorder diagnosed in the 12 months prior to CDAH2 (RR per kg/m^2^: 1.11 (0.94–1.30). In males, body weight satisfaction was not associated with episode of mood disorder or mood disorder diagnosed in the 12 months prior to CDAH2 (Table 3).

After adjusting for confounding factors in the base model, BMI at baseline did not significantly predict episode of anxiety in males (RR per kg/m^2^: 0.96, 95% CI: 0.87–1.07) or females (RR per kg/m^2^: 0.99, 95% CI: 0.94–1.04). Further adjustment for potential mediators, including body weight satisfaction, did not significantly change the associations between baseline BMI and episode of anxiety in both males (RR per kg/m^2^: 1.07, 95%CI: 0.76–1.50) and females (RR per kg/m^2^: 1.00, 95%CI: 0.93–1.09). The finding of no significant association between baseline BMI and episode of anxiety did not change when the analyses was limited to those diagnosed with anxiety in the previous 12 months of the CDAH2 in both males and females. The results are presented as Additional file 3.

## Discussion

This study of a cohort of young adults did not find a bidirectional relationship between anxiety and mood disorders and BMI. A history of mood disorder was significantly associated with an increase in BMI in males, while higher BMI at baseline was related to higher risk of mood disorders in females. These associations were mediated by dietary pattern and lifestyle factors, suggesting that health behaviour interventions may promote both physical and mental wellbeing. There was no significant association between history of anxiety and subsequent change in BMI or vice versa in both males and females.

### Association between BMI and mood disorder

This study adds to the relatively small number of prospective studies examining the temporal relationship between mood and anxiety disorders and change in BMI while controlling for the mediation effects of a wide range of variables. It is one of few studies to examine the effect of a wide range of potential confounding or mediating variables on these associations. While a bidirectional association between anxiety and BMI change is rarely reported, evidence regarding bidirectional association between mood disorder and BMI change is inconsistent [23, 24, 38]. There are several possible reasons for the inconsistent results among the studies including variation in the populations studied in terms of age at onset, duration of episodes, duration of study follow-up, method of mental disorder assessment, and severity of these disorders [21, 39, 40]. Recent evidence suggest that the unfavourable effect of depression on development of weight gain and the effect of weight gain on development of depression may be reinforced by follow-up time, suggesting that the associations between these disorders may be less apparent in studies with short follow-up [21]. Mood disorders with onset at younger ages tend to have a more unfavourable course and are associated with greater comorbidity than those with a late-onset [39]. Previous studies have also shown that younger adults are more likely to seek care for their mental illness, reducing the duration of an episode and recurrence rates [40–42], onset of mood disorder-related unhealthy lifestyle and comorbid conditions [40, 42].

The positive association between history of mood disorder and subsequent increase in BMI in males is consistent with the findings of some studies [43, 44]. The increase in BMI among males attributed to mood disorder is relatively small, although it could be a clinically significant increase in light of the wide range of adverse health effects with even a small increase in BMI above the normal range. For example, a one- kg/m^2^ increase in BMI raises heart failure risk by 17% [45], and type 2 diabetes by 15 and 11% in men and women, respectively, independent of subsequent weight changes [46].

There could be several explanations for our findings that the association between history of disorder and BMI was weaker in females than in males. Firstly, the statistical power to detect association is reduced due to variation introduced when applying methods to address large scale attrition in this cohort. The effect size estimate for females is smaller than that for males. This might be explained by the fact that females are more likely to report mental health disorders and seek mental health services [41], which may improve their quality of life and response to mood disorders [47]. According to the 2007 Australian health survey, more women (41%) with a 12-month mental disorder accessed services for mental health problems than men (28%) [48]. In the UK, more than 750,000 people were referred for counselling for anxiety and depression in 2012–2013, of whom 62% were women [41]. Women also tend to have a better coping strategies and favourable outcomes than men, which could possibly attenuate the potential BMI increase among women with history of mood disorder [49, 50]. In addition, evidence shows that depression-related unhealthy lifestyles, which in turn could lead to BMI change, are more common in males than in females [51].

We found that the association between baseline BMI and elevated risk of mood disorder was limited to females. This is consistent with findings from previous studies, which have demonstrated a relationship between BMI and subsequent risk of mood disorder in females but not in males [52, 53]. A longitudinal study of 4410 participants in the US found that overweight in adolescence was associated with higher odds of developing depressive symptoms (odds ratio:1.74) in later adulthood in females, but not in males [53]. One possible explanation is that, in women, higher BMI is related with negative body image perception and satisfaction, which in turn, is associated with higher risk of mood disorders [54]. According to previous studies, weight dissatisfaction is both common and has stronger effect on depression in middle-aged than in younger adults [55, 56].

Our findings on the mediating role of dietary pattern and health behaviours, for mood disorder and BMI, are consistent with the common factors associated with both conditions. Studies suggest that people living with mental health disorders are significantly more likely to engage in low levels of physical activity, more likely to smoke cigarettes and have poor diet quality [57, 58], which in turn are known to increase the risk of weight gain. On the other hand, weight gain increases risk of declining physical activity and diet quality over time [59]. Furthermore, increase in body weight has been shown to activate chronic low-grade inflammation and low-grade systemic inflammation contributes to the development of mental disorders [60, 61]. From a public health point of view, the mediating effect of physical activity and dietary patterns on the risk of BMI and mood disorder suggest that interventions targeting these risk factors may not only promote physical health but also improve mental health.

### Association between BMI and anxiety

The finding that history of anxiety was not associated with subsequent changes in BMI and vice-versa, is consistent with reports from previous studies [62–64], although positive associations have also been reported [38, 65, 66]. The reasons why previous studies have been inconsistent regarding the association between anxiety and change in BMI are not clear. One possible explanation could be the heterogeneity among the studies in terms of duration of follow-up, participant characteristics, and confounding variables. One previous study suggest that the association between depression and weight gain increases with increasing duration of follow-up, which could also be the case for anxiety [21]. Some of the studies that reported a positive association between weight or BMI and anxiety had longer duration of follow-up (up to 10 to 20 years) [38, 65]. Race may also play a role in the association between anxiety and weight gain. A population-based study among 103,557 residents of Olmsted County, Minnesota found that anxiety was significantly associated with obesity in Blacks but not in Asian or Hispanic groups [67]. However, data on race was not collected in our study, however, the majority of participants in the current study were Caucasian.

This study has some limitations. First, there was a substantial loss to follow-up from the original ASHFS random sample to the analysis sample. Study participants included in the present analyses were more likely to be employed, educated beyond high school, and have a healthier lifestyle [28]. However, inverse probability weighting, including variables that predicted non-response, was applied to minimize the effect of selection bias due to loss to follow-up. Participants were young-adults, and application of the findings to older populations in the community may be limited. Due to self-reporting, some misclassification of anxiety or mood disorder and under or over estimation of body weight is inevitable. However, both misclassification and under or over reporting are probably non-differential across the different study outcomes. Controlling for the confounding effect in the analyses may not entirely remove the confounding effect of some variables, such as diet and physical activity, which might have been measured without sufficient accuracy. Adjusting for change in predictor variables from baseline to follow-up may have potentially introduced reverse causation bias. However, the conclusions were consistent when the outcomes were BMI recorded at last follow-up and anxiety and mood disorder in the last 12 months. Furthermore, the multiple-testing issue may also be a limitation of this study. A substantial proportion of the participants were overweight or obese at baseline [33], which might have underestimated the change in BMI attributed to anxiety or mood disorders. Although correction for self-reporting error has been applied, the use of self-reported height and weight in some participants may have resulted in under-reporting of BMI.

Strengths of this study include its well-characterized cohort of younger adults, its use of adequate sample size, and its exploration of the mediation and confounding effect of a wide range of variables. Both lifestyle-related risk factors, and anxiety and mood disorders were measured using standardised data collection tools commonly used for epidemiological studies. The fact that the participants were younger individuals means that the findings are less likely to be confounded by chronic conditions. The findings were consistent when the analyses were restricted to those who have recent episode of anxiety or mood disorders.

## Conclusions

In a cohort of young adults, mood disorder before baseline is associated with elevated BMI in males, while baseline BMI increased the risk of developing mood disorder only in females. These associations were mediated by dietary pattern and lifestyle factors, suggesting that interventions promoting healthy lifestyle could contribute to reducing increase in BMI associated with mood disorder in males, and excess risk of mood disorder associated with BMI in females. There was no significant association between history of anxiety and subsequent change in BMI or vice versa in both males and females.

## Supplementary information

**Additional file 1.** Approaches to the data analyses.

**Additional file 2.** Effect of potential confounders or mediators on the associations between anxiety before baseline and weight at follow-up.

**Additional file 3.** Effect of potential confounders or mediators on the associations between BMI at baseline and episode of anxiety.

## Data Availability

Due to ethical restrictions, the data cannot be made publicly available. The datasets used during the current study are available from the corresponding author on reasonable request.

## Abbreviations

BMI: body mass index (kg/m2)
CDAH: Childhood Determinants of Adult Health
