# Supplementary material for: Association between depression, anxiety and weight change in young adults
Source: BMC Psychiatry. 2019 Dec 16;19:398. doi: 10.1186/s12888-019-2385-z (PMC6916239; doi:10.1186/s12888-019-2385-z)
Supplement: Supplementary file 1 — Additional file 1. Approaches to the data analyses. [file 12888_2019_2385_MOESM1_ESM.docx]

**Additional file 1:** Approaches to the data analyses

| Direction of analyses | Outcome variable | Model | Outputs |
| --- | --- | --- | --- |
| Episode of mood disorder before baseline to predict BMI at follow-up | BMI at follow-up | Multivariate linear regression | Table 2 |
| Episode of anxiety before baseline to predict BMI at follow-up | BMI at follow-up | Multivariate linear regression | *Supplementary file 2* |
| BMI at baseline to predict episode of mood disorder between baseline and follow-up | first episode of mood disorder since baseline | Log-binomial regression | Table 3 |
| BMI at baseline to predict mood disorder during the 12 months prior to the CDAH2 interview | Mood disorder during the 12 months prior to the CDAH2 interview | Log-binomial regression |  |
| BMI at baseline to predict episode of anxiety between baseline and follow-up | first episode of anxiety since baseline | Log-binomial regression | *Supplementary file 3* |
| BMI at baseline to predict anxiety during the 12 months prior to the CDAH2 interview | Anxiety during the 12 months prior to the CDAH2 interview | Log-binomial regression |  |

BMI: Body Mass Index (kg/m^2^)
