# Supplementary material for: Association between depression, anxiety and weight change in young adults
Source: BMC Psychiatry. 2019 Dec 16;19:398. doi: 10.1186/s12888-019-2385-z (PMC6916239; doi:10.1186/s12888-019-2385-z)
Supplement: Supplementary file 2 — Additional file 2. Effect of potential confounders or mediators on the associations between anxiety before baseline and weight at follow-up. [file 12888_2019_2385_MOESM2_ESM.docx]

**Additional file 2*:*** Effect of potential confounders or mediators on the associations between anxiety before baseline and BMI at follow-up

| **Variables** | Coefficient of history of anxiety after inclusion of covariate*  β (95%CI) | Total mediated effect |
| --- | --- | --- |
| **Males** |  |  |
| **Baseline measurements** |  |  |
| **Base model** | **0.16 (-0.45,0.76)** | - - - |
| C-reactive protein | 0.19 (-0.44,0.81) | -0.03 |
| Moderate or vigorous physical activity | 0.19 (-0.44,0.81) | -0.03 |
| Moderate or vigorous leisure time physical activity | 0.18 (-0.45,0.80) | -0.02 |
| Steps per day | 0.35 (-0.39,1.09) | -0.19 |
| Dietary guideline index | 0.28 (-0.43,0.99) | -0.12 |
| Adherence | 0.15 (-0.51,0.78) | 0.01 |
| Extra food consumption | 0.23 (-0.49,0.95) | -0.07 |
| Skipping breakfast | 0.15 (-0.49,0.79) | 0.01 |
| Parent to children | 0.19 (-0.44,0.82) | -0.03 |
| Fibrinogen | 0.18 (-0.45,0.81) | -0.02 |
| Antidepressant use | 0.19 (-0.44,0.83) | -0.03 |
| Smoking status | 0.18 (-0.44,0.81) | -0.02 |
| **Additional follow-up variables** |  |  |
| Change in moderate or vigorous physical activity | 0.15 (-0.48,0.79) | 0.01 |
| Change in moderate or vigorous leisure time physical activity | 0.16 (-0.48,0.80) | 0 |
| Change in steps per day | 0.53 (-0.29,1.35) | -0.37 |
| Dietary guideline index | 0.20 (-0.43,0.83) | -0.04 |
| Dietary adherence | 0.14 (-0.50,0.78) | 0.02 |
| Smoking status | 0.18 (-0.44,0.80) | -0.02 |
| **Change between baseline and follow-up** |  |  |
| Change in moderate or vigorous physical activity | -0.02 (-0.89,0.85) | 0.18 |
| Change in moderate or vigorous leisure time physical activity | 0.02 (-0.83,0.88) | 0.14 |
| Change in steps per day | 0.15 (-0.48,0.78) | 0.01 |
| Change in smoking status | 0.19 (-0.44,0.81) | -0.03 |
| Change in dietary Guideline Index | 0.17 (-0.46,0.80) | -0.01 |
| Parental change | 0.12 (-0.53,0.76) | 0.04 |
| Marital change | 0.19 (-0.44,0.82) | -0.03 |
| **Full model** | 0.19 (-0.44,0.82) | -0.03 |
| **Females** |  |  |
| **Base model** | **0.01 (-0.59-0.61)** | - - - |
| C-reactive protein | 0.16 (-0.48,0.79) | -0.15 |
| Moderate or vigorous physical activity | 0.16 (-0.48,0.80) | -0.15 |
| Moderate or vigorous leisure time physical activity | 0.14 (-0.49,0.77) | -0.13 |
| Steps per day | 0.47 (-0.28,1.21) | -0.46 |
| Dietary guideline index | 0.41 (-0.30,1.11) | -0.4 |
| Extra food consumption | 0.34 (-0.33,1.00) | -0.33 |
| Skipping breakfast | 0.15 (-0.49,0.79) | -0.14 |
| Parent to children | 0.16 (-0.47,0.79) | -0.15 |
| Fibrinogen | 0.16 (-0.48,0.80) | -0.15 |
| Antidepressant use | 0.19 (-0.44,0.82) | -0.18 |
| Smoking status | 0.17 (-0.44,0.79) | -0.16 |
| **Additional follow-up variables** |  |  |
| Moderate or vigorous physical activity | 0.17 (-0.46,0.81) | -0.16 |
| Moderate or vigorous leisure time physical activity | 0.19 (-0.44,0.82) | -0.18 |
| Steps per day | 0.54 (-0.16,1.25) | -0.53 |
| Dietary guideline index | 0.20 (-0.47,0.87) | -0.19 |
| Dietary adherence | 0.16 (-0.48,0.81) | -0.15 |
| Smoking status | 0.21 (-0.41,0.82) | -0.2 |
| **Change between baseline and follow-up** |  |  |
| Change in moderate or vigorous physical activity | 0.47 (-0.20,1.15) | -0.46 |
| Change in moderate or vigorous leisure time physical activity | 0.51 (-0.17,1.19) | -0.5 |
| Change in steps per day | 0.20 (-0.44,0.85) | -0.19 |
| Change in smoking status | 0.14 (-0.48,0.76) | -0.13 |
| Change in dietary Guideline Index | 0.16 (-0.48,0.79) | -0.15 |
| Parental change | 0.16 (-0.47,0.80) | -0.15 |
| Marital change | 0.16 (-0.48,0.80) | -0.15 |
| **Full model** | 0.16 (-0.48,0.80) | -0.15 |

CI: confidence interval, β-adjusted and weighted effect of anxiety on BMI at follow-up Base model adjusted for: Bassline BMI (kg/m2), age, education, duration of follow-up, history of cardiometabolic disease, Social Support index, self-rated physical health status, openness and extraversion. *Covariates, associated with anxiety based on a univariate models (at p<0.1) were added at a time to the base model to test for mediating.
