# Supplementary material for: Association between depression, anxiety and weight change in young adults
Source: BMC Psychiatry. 2019 Dec 16;19:398. doi: 10.1186/s12888-019-2385-z (PMC6916239; doi:10.1186/s12888-019-2385-z)
Supplement: Supplementary file 3 — Additional file 3. Effect of potential confounders or mediators on the associations between BMI at baseline and episode of anxiety. [file 12888_2019_2385_MOESM3_ESM.docx]

**Additional file 3:** Effect of potential confounders or mediators on the associations between BMI at baseline and episode of anxiety

| **Variables** | First episode of anxiety since baseline (vs. those with no history) | PERM | Episode of anxiety 12 leading to CDAH2 (vs those with no history) | PERM |
| --- | --- | --- | --- | --- |
|  | **Adjusted RR (95% CI)** after inclusion of covariate |  | **Adjusted RR (95% CI)** after inclusion of covariate |  |
| **Males** |  |  |  |  |
| Base model | 0.96 (0.87,1.07) | - - - | 0.95 (0.87,1.03) | - - - |
| C-reactive protein | 0.99 (0.89,1.10) | 75 | 0.96 (0.87,1.05) | 20 |
| Dietary guideline index | 1.00 (0.83,1.21) | 100 | 0.93 (0.78,1.11) | -40 |
| Antidepressant use | 1.03 (0.95,1.11) | 175 | 0.96 (0.81,1.14) | 20 |
| Smoking status | 1.01 (0.92-1.10) | 125 | 0.97 (0.89,1.05) | 40 |
| Fibrinogen | 1.02 (0.93,1.12) | 150 | 0.96 (0.81,1.14) | 20 |
| Moderate or vigorous physical activity | 1.02 (0.87,1.25) | 150 | 0.92 (0.78,1.08) | -60 |
| Steps per day | 1.02 (0.85,1.23) | 150 | 0.90 (0.77,1.07) | -100 |
| Extra food consumption | 1.02 (0.91,1.16) | 150 | 0.97 (0.89,1.06) | 40 |
| Smoking status2 | 1.02 (0.94,1.11) | 150 | 0.91 (0.77,1.08) | -80 |
| Moderate or vigorous physical activity 2 | 1.01 (0.92,1.11) | 125 | 0.97 (0.89,1.06) | 40 |
| Dietary guideline index 2 | 1.01 (0.92,1.11) | 125 | 0.90 (0.76,1.07) | -100 |
| Weight satisfaction | 1.03 (0.81,1.32) | 175 | 0.99 (0.88-1.11) | 80 |
| Takeaway food consumption | 1.04 (0.88,1.23) | 200 | 0.99 (0.91-1.08) | 80 |
| Dietary adherence | 1.07 (0.76,1.50) | 275 | 0.98 (0.90-1.06) | 60 |
| **Full model** | **1.07 (0.76,1.50)** | **275** | **0.98 (0.90-1.06)** | **60** |
| **Females** |  |  |  |  |
| Base model | 0.99 (0.94,1.04) | - - - | 1.01 (0.98,1.04) | - - - |
| C-reactive protein | 0.99 (0.94,1.05) | 0 | 1.02 (0.98,1.06) | -100 |
| Dietary guideline index | 1.01 (0.96,1.07) | -200 | 1.04 (0.97,1.13) | -300 |
| Smoking status | 1.02 (0.96-1.08) | 300 | 1.02 (0.98,1.06) | -100 |
| Antidepressant use | 1.00 (0.93,1.07) | 100 | 1.03 (0.94,1.13) | -200 |
| Fibrinogen | 0.98 (0.91,1.05) | -100 | 1.01 (0.96,1.06) | 0 |
| Moderate or vigorous physical activity | 0.96 (0.84,1.11) | -300 | 1.03 (0.93,1.13) | -200 |
| Smoking status2 | 1.00 (0.93,1.07) | 100 | 1.02 (0.93,1.13) | -100 |
| Moderate or vigorous physical activity2 | 0.98 (0.92,1.05) | -100 | 1.03 (0.94,1.12) | -200 |
| Dietary guideline index | 0.96 (0.84,1.11) | -300 | 1.02 (0.92,1.13) | -100 |
| Steps per day | 1.00 (0.92,1.09) | 100 | 1.02 (0.92,1.13) | -100 |
| Extra food consumption | 1.00 (0.85,1.18) | 100 | 1.01 (0.97,1.06) | 0 |
| Weight satisfaction | 1.03 (0.96,1.11) | 400 | 1.00 (0.94-1.06) | 100 |
| Takeaway food consumption | 1.01 (0.93,1.09) | 200 | 1.04 (0.95-1.13) | -300 |
| Dietary adherence | 1.00 (0.93,1.09) | 100 | 1.01 (0.97-1.06) | 0 |
| **Full model** | **1.00 (0.93,1.09)** | **100** | **1.01 (0.97-1.06)** | **0** |

CI, confidence interval PERM, Percentage of excess risk mediated; RR, relative risk; Base model adjusted for: Age, education, duration of follow-up, marital status, self-reported physical health status, history of cardiometabolic diseases, and use of oral contraceptive (in women); PERM-percentage of excess risk explained by the mediator.
